# The Potential of Diffusion-Based Near-Infrared Image Colorization

**DOI:** 10.3390/s24051565

**Published:** 2024-02-28

**Authors:** Ayk Borstelmann, Timm Haucke, Volker Steinhage

**Affiliations:** 1 Institute of Computer Science IV, University of Bonn, Friedrich-Hirzebruch-Allee 8, 53115 Bonn, Germany; haucke@cs.uni-bonn.de; 2 Computer Science & Artificial Intelligence Laboratory, Massachusetts Institute of Technology, 32 Vassar St., Cambridge, MA 02139, USA

**Keywords:** near-infrared, diffusion models, camera trapping, unpaired dataset, neural networks, machine learning

## Abstract

Camera traps, an invaluable tool for biodiversity monitoring, capture wildlife activities day and night. In low-light conditions, near-infrared (NIR) imaging is commonly employed to capture images without disturbing animals. However, the reflection properties of NIR light differ from those of visible light in terms of chrominance and luminance, creating a notable gap in human perception. Thus, the objective is to enrich near-infrared images with colors, thereby bridging this domain gap. Conventional colorization techniques are ineffective due to the difference between NIR and visible light. Moreover, regular supervised learning methods cannot be applied because paired training data are rare. Solutions to such unpaired image-to-image translation problems currently commonly involve generative adversarial networks (GANs), but recently, diffusion models gained attention for their superior performance in various tasks. In response to this, we present a novel framework utilizing diffusion models for the colorization of NIR images. This framework allows efficient implementation of various methods for colorizing NIR images. We show NIR colorization is primarily controlled by the translation of the near-infrared intensities to those of visible light. The experimental evaluation of three implementations with increasing complexity shows that even a simple implementation inspired by visible-near-infrared (VIS-NIR) fusion rivals GANs. Moreover, we show that the third implementation is capable of outperforming GANs. With our study, we introduce an intersection field joining the research areas of diffusion models, NIR colorization, and VIS-NIR fusion.

## 1. Introduction

**Figure 1:**
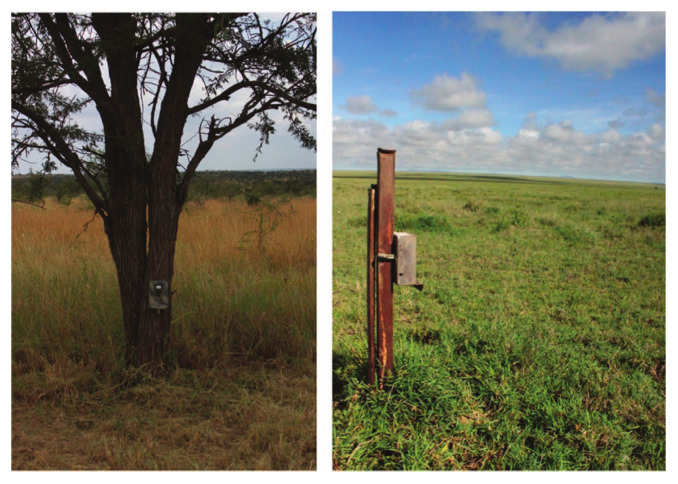
Camera trapping for the Snapshot Serengeti dataset. ©Swanson, Kosmala, Lintott, Simpson, Smith, and Packer [8]. Licensed under a Creative Commons Attribution 4.0 License.

For wildlife monitoring, typically, camera traps are used (see Figure 1). Camera traps show several advantages for wildlife monitoring:

1. Camera traps deliver permanent documentation records of date, location, and species.
2. These camera-trap-based documentation records allow for estimations of animal populations [1] and movements of animals and herds [2].
3. Camera traps can record animal behavior [3].
4. Using invisible infrared flashlights, camera traps work non-invasive and, therefore, have no disturbing effects on animal behavior.
5. Camera traps work efficiently for several weeks [4].
6. Camera trapping allows for synergies between expert and citizen science [5].
7. Images and video clips can be used for education, promotion, and funding acquisition [6,7].

### 1.1. Problem Statement

During daylight, normal cameras succeed in capturing detailed images. But at dawn or during nighttime, near-infrared (NIR) cameras or normal cameras with incandescent lighting are necessary. Near-infrared light has a wavelength between 750 nm and 1400 nm, which mostly lies outside the visible spectrum (380 nm–780 nm) [9]. Because of that, NIR cameras offer a significant advantage over conventional cameras using incandescent lighting. Incandescent light flashes are visible to animals and may frighten them, leading to animals avoiding the camera location afterward, which in turn would corrupt statistical estimates like population or migration estimations based on the numbers and frequencies of observed animals. Near-infrared light is not visible to animals and thus cannot scare them.

But on the other hand, NIR images appear as grayscale images that do not conform to the human visual spectrum because they lack colors and color textures. Therefore, it can be difficult to perceive the details of observed scenes in NIR images [10]. This discrepancy between NIR and colored images constitutes the domain gap between near-infrared and colored images.

Additionally, in recent years the combination of camera trapping and of artificial intelligence (AI), especially of deep learning approaches, has emerged as a breakthrough in the field of wildlife research and conservation [3,7,11,12]. However, many deep learning approaches are trained for and can benefit from colored images [13] like humans do [10]. This raises the question if deep learning approaches can also benefit from such artificially colored images. We evaluate and discuss if this is the case in Section 4.2.

### 1.2. Contribution

In this study, we propose the automated conversion of NIR images to colored RGB images

- to derive detail-rich images providing color and texture without scaring animals;
- to gain compatibility with and benefits for existing monitoring systems;
- to improve human comprehension of camera trap data.

#### 1.2.1. Colorizing NIR Images—Luminance and Chrominance

It is important to note that colorizing NIR images is closely related to the colorization of grayscale images, but it differs in one crucial property: the luminance (i.e., the amount of light that is reflected off an object) of grayscale images captured in the visible spectrum is the same as the luminance of colored images. Therefore, only chrominance (i.e., the color component) must be estimated by image colorization systems. However, the objects’ reflection properties of NIR light differ from those of visible light. Consequently, due to the differing luminance between RGB and NIR images, conventional image colorization techniques cannot be applied as-is.

#### 1.2.2. Colorizing NIR Images—Paired vs. Unpaired Image Translation

Supervised solutions for this problem exist but require NIR and RGB image pairs with pixel-to-pixel registration and temporal synchronization. For each given NIR image, the corresponding RGB image must have the same information on the pixel locations on both images, and both images must be captured simultaneously to account for motion. However, because many wildlife datasets involve the use of NIR cameras for nighttime and regular cameras for daytime images, it is rare to find paired datasets. This results in unpaired image translation to which unsupervised learning techniques must be applied.

#### 1.2.3. Colorizing NIR Images—GAN-Based Approaches vs. Diffusion Models

Current state-of-the-art approaches leverage generative adversarial networks (GANs) to solve this unpaired image translation problem. For example, Mehri and Sappa [14] proposed a GAN-based approach specifically designed for the task of colorizing NIR images (cf. Section 1.3). However, GANs are known for their unstable training manifesting in mode collapse and hallucinations [15].

Recently, advances in denoising diffusion probabilistic models (short: diffusion models or DDPM) [16] showed superiority over GANs in various other image translation tasks [17,18]. Their training is more stable, while simultaneously a higher sample diversity is observed. This suggests that diffusion models could also influence NIR colorization positively.

#### 1.2.4. Colorizing NIR Images: Refined Contribution

To the best of our knowledge, this study proposes the first automated conversion of NIR images to colored RGB images utilizing diffusion models. The novelty of our approach lies in discovering the key property necessary to let a diffusion model generate realistic images, i.e., the appropriate translation of the NIR image intensities into color intensities.

Thereby, we provide a generic approach in terms of a framework where we first abstract the translation of the intensities to allow for implementations and evaluations of different approaches to intensity translations.

The framework is based on iterative latent variable refinement (ILVR) [19] but comes with the following novel methodical improvements to specialize for near-infrared colorization:

- Replacing the low-pass filter as latent variable refinement technique;
- Differentiating into merging chrominance and merging intensity instead;
- Abstracting the intensity translation.

Based on the abstraction of the intensity translation, we provide and evaluate three different specific implementations. The evaluation of these shows that even the deployment of trivial algorithms inspired by insights gained in the VIS-NIR fusion research field [20] can achieve Fréchet inception distances (FIDs) close to GAN baselines. This employs a connection between the research fields of diffusion models, near-infrared colorization, and visible near-infrared fusion. Finally, we show that our framework is capable of outperforming a GAN baseline, revealing the potential of diffusion-based NIR colorization.

### 1.3. Related Work

NIR colorization has many applications in addition to wildlife monitoring, e.g., in driver assistance systems or surveillance cameras. As a result, computer vision researchers have studied image colorization during the last decades, and thus, many solutions exist. Solutions for NIR colorization are divided into paired and unpaired image translation problems, and thus, supervised and unsupervised learning techniques are applied. For paired image translation, pixel-to-pixel registration and temporal synchronization are required for each image pair, which adds additional challenges.

Limmer and Lensch [21] used a dataset acquired by a specialized multi-CCD camera that ensures the requirement of the image pair. As a translation mechanism, Limmer and Lensch [21] proposed to use deep multiscale convolutional neural networks (CNN). In pre-processing, a normalized image pyramid is constructed from the NIR input, and in post-processing, the CNN output is enriched with details from the input image. In later work, Dong et al. [22] introduced an S-shape network consisting of one U-Net-based encoder “ColorNet” and a shallow network that generates an edge loss function “EdgeNet”. Dong et al. [22] created a pixel-to-pixel registered dataset using geometric transformations and feature-based correspondence methods.

#### 1.3.1. GAN-Based Approaches

GAN-based methods have established themselves as a powerful approach to unpaired image translation, which can be used for NIR image colorization without the need for paired training data. This is especially useful if such data cannot be obtained, for example, due to dataset limitations. By default, GANs tend to lose the content of the input image. CycleGAN, and an architecture proposed by Zhu et al. [23] uses a cycle consistency loss between the input and the generated image to solve that problem. It uses ResNet [24] as the generator network [23]. Gao et al. [13] trained CycleGAN on a wildlife dataset and showed improved recognition results on the generated images compared to the NIR images. Mehri and Sappa [14] proposed a version of CycleGAN, specifically designed for the task of colorizing NIR images that incorporates enhanced loss functions and utilizes U-Net as a generator. Because of this, we use this CycleGAN of Mehri and Sappa [14] as a GAN baseline for our research.

We use the GAN DeOldify [25] as a second reference method since it is trained on a large dataset.

#### 1.3.2. Diffusion Models

More recently, diffusion models have advanced. First suggested by Sohl-Dickstein et al. [26], diffusion models are neural networks that gradually remove noise from signals. Simultaneously to Sohl-Dickstein et al. [26], Song and Ermon [27] introduced and studied score matching as a way of estimating the given data distribution using its gradients while sampling with Langevin dynamics [28]. Later, Ho et al. [16] first found the connection between diffusion models and score-based models and leveraged this to simplify the training objective of a variational lower bound. They introduced denoising diffusion probabilistic models (DDPM), which is considered a milestone in the development of diffusion models.

Song et al. [18] further analyzed the connection between score matching with Langevin dynamics and diffusion models, proposed a unified framework using the stochastic differential equation, and showed that both DDPM [16] and their previous work [27] can be considered a specialized formulation of it. Further, they introduced deterministic samplers using ordinary differential equations that allow likelihood computation and deterministic latent codes. Most important for us, through their formulation, they derive a conditional sampling method that only uses an unconditional model to control the generation at inference time. This allows applications to image imputation and grayscale colorization, which we base our work on.

Dhariwal and Nichol [17] were the first to outperform GANs in image generation with several architectural improvements. Additionally, they introduced classifier guidance. This sampling method uses unconditional diffusion models and only a classifier during inference to achieve class conditional sampling. With this, they provided a conditional sampling method inspired by Song et al. [18] for DDPMs.

Saharia et al. [29] trained a multi-task image-conditional diffusion model with application to grayscale colorization. In contrast to our approach, they used supervised learning to train a conditional diffusion model and, therefore, required a pair dataset at training time.

Choi et al. [19] leveraged an unconditional diffusion model and iteratively refined the current sample (ILVR). By this, they achieved conditional sampling. Our framework iteratively refines the latent variable by enriching it with information from the near-infrared image. Therefore, we consider our framework heavily based on ILVR’s key algorithm. However, ILVR enriches the input image with low-frequency information from the input image. Binding the low frequencies of the generated image to those of the near-infrared image does not lead to colorization; instead, it just results in grayscale images with similar contours to the given image.

Zhao et al. [30] suggested an energy term for diffusion models, describing the similarity and steering the sampling using its gradient. Their energy is divided into domain-independent energy and domain-specific energy. The domain-independent energy ensures similarity to the input image, while the domain-specific energy ensures realism in the output domain [30]. They employed a low-pass filter as a domain-independent extractor, which NIR colorization does not benefit from. However, it is mentioned that different domain-independent extractors are feasible.

Furthermore, research from the visible-infrared fusion field influences our work. VIS-NIR fusion focuses on enhancing RGB images with NIR images. As our approach can be considered iteratively fusing visible and infrared images, we borrow insights from this field of research. Sharma et al. [20] studied and compared comprehensively multiple visible-infrared fusion techniques. One common similarity between many of the compared methods is that the near-infrared intensities are combined with visible intensities at different scales and with the chrominance of the visible-light image [20,31,32]. We evaluate this principle as a strategy for enriching the latent variable in our framework.

We develop a novel NIR colorization approach to images leveraging the recent advances of diffusion models. Focus is placed on the unpaired image translation because NIR-RGB image pairs are often hard to obtain. We only need to train an unconditional diffusion model in the target domain. Our framework is based on ILVR [19] and abstracts the intensity translation. We present three implementations of this framework. First, we use NIR intensities, which are effectively equivalent to the grayscale colorization of Song et al. [18]. Next, we utilize the connection to VIS-NIR fusion [20] and present an implementation based on fusioning high frequencies of near-infrared images with low frequencies of the colored image. Finally, we show the potential of our method by using CycleGAN itself as an intensity translator.

## 2. Materials and Methods

Denoising diffusion probabilistic models (DDPMs), as introduced by Ho et al. [16], are recent advances in the field of image generation. We provide a theoretical background for this architecture in Section 2.1 and show how an unconditional diffusion model can be used to sample with inputs. Iterative Seeding, our framework leveraging these diffusion models for colorization, is presented in Section 2.2, and two implementations are presented in Section 2.2.1 and Section 2.2.2.

For developing a diffusion near-infrared colorization approach, a dataset containing NIR and colored images from similar settings is required. We choose the Snapshot Serengeti dataset originating in the Serengeti National Park in Tanzania. It consists of 7.1 million images captured over the course of seven seasons of the Snapshot Serengeti Project [8]. There are 61 labeled species, while approximately 76% of the images are labeled as empty. For training and evaluation, we create a subset of the dataset consisting of 10,000 images (5000 NIR and 5000 colored images). We partition this subset into an 8000-image train dataset and two separate datasets for testing and validation, each consisting of 1000 images. Furthermore, night images are chosen only because they align with the application context of near-infrared colorization, and the network does not implicitly learn to translate night images to day images.

### 2.1. Background

A diffusion process, consisting of *T* time steps, describes how noise is added to an image. $x_{0}$ denotes the original image and $x_{T}$ the final noised image. *T* is chosen, so that $x_{T}$ follows an isotropic Gaussian distribution [16].

With $q(x_{t}|x_{t-1})$ we denote the forward process, which describes the distribution of $x_{t}$ given a less noised $x_{t-1}$. The forward process gradually adds Gaussian noise to the image determined by the variance schedule $\beta_{1},\cdots ,\beta_{T}$ [16] (Equation (1)).

(1) $$\begin{array}{cc} q(x_{t}|x_{t-1}):= & N(x_{t};\sqrt{1-\beta_{t}}x_{t-1},\beta_{t}I) \end{array}$$

To sample $x_{t}∼q\left(x_{t}|x_{0}\right)$, repeated sampling is not necessary because a closed form can be derived (Equation (2)), where $\alpha_{t}:=1-\beta_{t}$ and ${\bar{\alpha }}_{t}:=\prod_{s=1}^{t}\alpha_{s}$ [16].

(2) $$\begin{array}{cc} q(x_{t}|x_{0}) & =N(x_{t};\sqrt{{\bar{\alpha }}_{t}}x_{0},\left(1-{\bar{\alpha }}_{t}\right)I) \end{array}$$

The reverse process describes how DDPMs operate. Initially, a sample is drawn from the prior distribution $q(x_{T})$, which is nearly an isotropic Gaussian, therefore $x_{T}∼N\left(0,I\right)$. Then we gradually denoise our sample using $q(x_{t-1}|x_{t})$ until t=0. Because $q(x_{t-1}|x_{t})$ is not trivially obtainable without knowing the data distribution, we leverage a neural network $p_{\theta }$ to approximate it. θ denotes the parameters of the network. If $\beta_{t}$ is small enough, $p_{\theta }\left(x_{t-1}|x_{t}\right)$ will also be Gaussian (Equation (3)) [16]. Note that Ho et al. [16] fix the variance of the reverse process using $\sigma_{t}^{2}I$ with either $\sigma_{t}^{2}=\beta_{t}$ or $\sigma_{t}^{2}={\tilde{\beta }}_{t}$.

(3) $$\begin{array}{cc} p_{\theta }\left(x_{t-1}|x_{t}\right) & :=N(x_{t-1};\mu_{\theta }\left(x_{t},t\right),\sigma_{t}^{2}I) \end{array}$$

Ho et al. [16] choose to parameterize $\mu_{\theta }\left(x_{t},t\right)$ as follows, where $\epsilon_{\theta }$ is a function to predict ϵ given $x_{t}$ (Equation (4)).

(4) $$\mu_{\theta }\left(x_{t},t\right)=\frac{1}{\sqrt{\alpha_{t}}}\left(x_{t}-\frac{\beta_{t}}{\sqrt{1-{\bar{\alpha }}_{t}}}\epsilon_{\theta }\left(x_{t},t\right)\right)$$

Furthermore, Ho et al. [16] suggest a simplified loss (Equation (5)), which uses the μ-parameterization (Equation (4)).

(5) $$L_{simple}\left(\theta \right):=E_{t∼\left[1,T\right],x_{0},\epsilon_{t}}\left[\left|\right|\epsilon_{t}-\epsilon_{\theta }\left(\sqrt{{\bar{\alpha }}_{t}}x_{0}+\sqrt{1-{\bar{\alpha }}_{t}}\epsilon ,t\right){\left|\right|}^{2}\right]$$

In terms of network architecture, diffusion models usually employ the U-Net architecture for learning the noise $\epsilon_{\theta }\left(x_{t},t\right)$ [14,16,17,33]. The U-Net takes the current noised image $x_{t}$ as input and aims to produce the noise that should be removed. We use the refined U-Net architecture from Dhariwal and Nichol [17] which included global attention blocks and embedding of the timestep *t*.

Our application context of NIR colorization is not able to benefit from the unconditional sampling as derived up until now. We need a method to condition the diffusion model on the given NIR image at inference time (unpaired translation). This is equivalent to sampling from a conditional distribution $p(x_{t-1}|x_{t},c)$, where c denotes the NIR image. Similar to Choi et al. [19], we can utilize the unconditional diffusion model, sample ${\tilde{x}}_{t-1}∼p_{\theta }\left({\tilde{x}}_{t}|x_{t}\right)$, and refine ${\tilde{x}}_{t-1}$ to be congruent to the condition c and obtain $x_{t}$. Choi et al. [19] use a low-pass filter to maintain similarity to the input image without restricting the sampling procedure. This is not suitable for NIR colorization because the low-pass filter would revert the colorization process performed by the diffusion model.

### 2.2. Iterative Seeding

Colorization can also be considered as a specialized form of image imputation. As Song et al. [18] showed. Image imputation is the task of restoring lost parts of an image congruent with the known areas of the image. In the case of grayscale colorization, the known part is the intensity, while unknown is the chrominance, which itself can be decomposed into hue and saturation.

As the intensity is not directly known in near-infrared colorization, we take an abstraction approach. In each iteration, we draw a sample ${\tilde{x}}_{t-1}$ from the diffusion model given $x_{t}$ using $p_{\theta }\left({\tilde{x}}_{t-1}|x_{t}\right)$. Simultaneously, we diffuse our input image $y_{0}$ to the timestep t−1 using $q(y_{t-1}|y_{0})$. We then decompose both images into their intensity parts ${\tilde{x}}_{t-1}^{I}$, $y_{t-1}^{I}$ and the chrominance parts ${\tilde{x}}_{t-1}^{C}$, $y_{t-1}^{C}$ using DecoupleIntensity without loss of information. A translation function TranslateIntensity enriches the intensity ${\tilde{x}}_{t-1}^{I}$ of our current sample with the near-infrared intensities $y_{t-1}^{I}$, returning a new visible-light intensity $x_{t-1}^{I}$ for the timestep t−1. This intensity $x_{t-1}^{I}$ is then combined with the chrominance of the sample ${\tilde{x}}_{t-1}^{C}$ and transformed back into the RGB domain using CoupleIntensity to obtain $x_{t-1}$.

The approach to only sample the intensity for colorization was first proposed by Song et al. [18]. But we do not implement this using a stochastic differential equation. Our procedure is more similar to Choi et al. [19]’s iterative latent variable refinement, and thus, we call this framework Iterative Seeding. In Algorithm 1, we demonstrate the code for our framework.
**Algorithm** **1** Iterative Seeding**Require:** Reference gray-scale image $y_{0}$, function TranslateIntensity returning intensities of visible light given the current near-infrared intensity and the current visible-light intensity    $x_{T}∼N\left(0,I\right)$    **for** t=T,⋯,1 **do**        $y_{t-1}∼q\left(y_{t-1}|y_{0}\right)$        ${\tilde{x}}_{t-1}∼p_{\theta }\left({\tilde{x}}_{t-1}|x_{t}\right)$        $y_{t-1}^{I},y_{t-1}^{C}=$ DecoupleIntensity($y_{t-1}$)        ${\tilde{x}}_{t-1}^{I},{\tilde{x}}_{t-1}^{C}=$ DecoupleIntensity(${\tilde{x}}_{t-1}$)        $x_{t-1}^{I}=$ TranslateIntensity($y_{t-1}^{I},{\tilde{x}}_{t-1}^{I}$)        $x_{t-1}=$ CoupleIntensity($x_{t-1}^{I}$,${\tilde{x}}_{t-1}^{C}$)    
**return** $x_{0}$

DecoupleIntensity and CoupleIntensity can theoretically be any invertible transformation where the intensity is decoupled from the color information. Many color spaces fulfill this property, e.g., HSI, HSV, LAB, and YCbCr, but we found empirically that transforming the RGB image using an orthogonal matrix with one dimension in resulting space being the intensity, like Song et al. [18] did, to give the best results.

Therefore, we search for a matrix $C\in R^{3\times 3}$ such that for any RGB pixel $p=\left(r,g,b\right)\in R^{3}$ and a fixed scalar a∈R the requirements of Equation (6) are fulfilled.

(6) $$\begin{array}{cc} p^{\prime } & =p\cdot C\Rightarrow p_{1}^{\prime }=a\cdot \left(r+g+b\right)\\ p & =\left(p\cdot C\right)\cdot C^{T} \end{array}$$

A matrix fulfilling those requirements can be obtained from solving a system of equations or using QR decomposition. We derive the matrix *C* as Equation (7). Note that Song et al. [18] use a different matrix as solution for this problem.

(7) $$\begin{array}{cc} C & =\left(\begin{array}{ccc} \frac{1}{\sqrt{3}} & -\frac{1}{2}-\frac{1}{2\sqrt{3}} & \frac{1}{2}-\frac{1}{2\sqrt{3}}\\ \frac{1}{\sqrt{3}} & \frac{1}{2}-\frac{1}{2\sqrt{3}} & -\frac{1}{2}-\frac{1}{2\sqrt{3}}\\ \frac{1}{\sqrt{3}} & \frac{1}{\sqrt{3}} & \frac{1}{\sqrt{3}} \end{array}\right)\approx \left(\begin{array}{ccc} 0.577 & -0.789 & 0.211\\ 0.577 & 0.211 & -0.789\\ 0.577 & 0.577 & 0.577 \end{array}\right) \end{array}$$

TranslateIntensity is the central component of our framework as it is an abstract function that is implemented in our study with increasing complex functions (cf. Section 2.2.1, Section 2.2.2, Section 3.2 and Section 3.3). Thereby, TranslateIntensity with its different implementations influences greatly the performance of the colorization. It should integrate information from both the near-infrared intensity $y_{t-1}^{I}$ and the approximation of visible-light intensity ${\tilde{x}}_{t-1}^{I}$ and produce an improved approximation $x_{t-1}^{I}$ of visible-light intensity. We note that this method is strongly related to the research domain of near-infrared and visible-light fusion (VIS-NIR fusion) since it practically fuses the near-infrared intensity $y_{t-1}^{I}$ and the visible-light intensity ${\tilde{x}}_{t-1}^{I}$. So, it is obvious to use approaches from this domain to implement this method. In Section 2.2.1 and Section 2.2.2, we present two different implementations of this method and evaluate them in Section 3.

#### 2.2.1. Near-Infrared Intensities

One simple strategy to implement TranslateIntensity is to directly use the near-infrared intensities. In that case, TranslateIntensity is the identity function for $y_{t-1}^{I}$ (Equation (8)). This implementation of our framework fixates the intensity of the output color to the near-infrared while giving the diffusion model just the freedom to sample the chrominance. Using this method does not reflect any near-infrared properties. It could also be applied to grayscale colorization and is conceptionally equivalent to Song et al. [18] colorization variant.

Leaving the diffusion model no freedom to generate intensity-related changes suggests a weakness of this implementation for near-infrared colorization. In Section 3.1, we evaluate this hypothesis. Compared to other implementations of our framework, this implementation performs the worst in terms of FID, confirming our hypothesis.

(8) $$\begin{array}{c} \mathrm{TRANSLATEINTENSITY}\left(y_{t-1}^{I},{\tilde{x}}_{t-1}^{I}\right):=y_{t-1}^{I} \end{array}$$

#### 2.2.2. High-Pass Filtering

A more refined approach to just using the near-infrared intensities is inspired from the VIS-NIR fusion domain. One key insight for fusioning NIR and visible-light images, is to combine the NIR image with intensities from the visible-light image at different scales [20].

A simple implementation of this concept is using the high frequencies of the near-infrared image and combining them with the low frequencies of the visible light’s intensity. In practise, a simple Gaussian filter $G\in R^{k\times k}$ (Equation (9)) can obtain the low frequencies [34], and the high frequencies are obtained by subtracting the low frequencies from the image (Algorithm 2).

(9) $$\begin{array}{c} G_{\sigma }\left(u,v\right)=\frac{1}{2\pi \sigma^{2}}\exp\left(-\frac{\left(u^{2}+v^{2}\right)}{2\sigma^{2}}\right) \end{array}$$

**Algorithm** **2** Implementation using High-Pass Filtering
**Require:** Gaussian filter $G_{\sigma }$    **procedure** TranslateIntensity($y_{t-1}^{I},{\tilde{x}}_{t-1}^{I}$)        ${\tilde{x}}_{t-1}^{I_{L}}={\tilde{x}}_{t-1}^{I}*G_{\sigma }$        ${\tilde{x}}_{t-1}^{I_{H}}={\tilde{x}}_{t-1}^{I}-{\tilde{x}}_{t-1}^{I_{L}}$        $y_{t-1}^{I_{L}}=y_{t-1}^{I}*G_{\sigma }$        $y_{t-1}^{I_{H}}=y_{t-1}^{I}-y_{t-1}^{I_{L}}$        $x_{t-1}^{I}={\tilde{x}}_{t-1}^{I_{L}}+y_{t-1}^{I_{H}}$        **return** $x_{t-1}^{I}$


With this implementation of our framework, iteratively the generated image is enriched with details of the near-infrared image. Thus, the diffusion model is only restricted to using the high frequencies of the near-infrared image and is free to sample low frequencies and the chrominance. This suggests a better performance can be reached than when only sampling the chrominance. On the other hand, this could also lead to a more difficult generation task, as less information is given.

We apply and evaluate this approach in Section 3.2 and confirm that this implementation performs better, through more freedom for the generator.

Further we investigate the influence of different standard deviations σ controlling the degree of information given in Section 3.2. We discover that this hyperparameter does affect the content preservation and realism of the generated images (Section 3.2).

## 3. Experimental Results

Our application context of near-infrared colorization lies in closing the gap between NIR and RGB images as inputs for deep learning systems, improving object recognition results by enriching the input with more information, and providing more familiar images to human users.

Since this is an unpaired image translation problem, classic solutions for quantitative evaluation, such as the difference between the absolute intensity values or SSIM [35], cannot be applied. To assess the realism of our results, we calculate the distance between the test dataset, consisting of real images, and our generated images using the Fréchet inception distance (FID) [36]. The FID acts as a distance between two unpaired image sets and is calculated on a classification network’s feature abstraction of images [36]. CleanFID is conceptually equivalent to the FID but comes with regularization techniques to make it more robust in terms of distortions, blurring, and compression artifacts [37]. Because of this, we use CleanFID instead of the regular FID. Furthermore, we evaluate our results with two blind/no-reference image quality assessment metrics NIQE [38] and NRQM [39]. Both score the realism images without the reference-image-based properties of the image [38,39].

Initially, an unconditional diffusion model is trained using the improved architecture from Dhariwal and Nichol [17]. All hyperparameters are taken from Dhariwal and Nichol [17] as well but adjusted for less powerful hardware. For both training and inference, an image resolution of 128×128 is used. We use a U-Net with five encode and five decode blocks, where each encode and decode block consists of two residual blocks [17]. Attention blocks are applied at the resolutions of 32×32, 16×16, and 8×8 like Dhariwal and Nichol [17] did. The noise schedule is divided into 1000 linear steps. We train with a batch size of 256 and a learning rate of $10^{-4}$ for 200 K iterations.

For CycleGAN, we train and evaluate a U-Net for the image resolution of 128×128. We train with a hyperparameter-optimized generator learning rate of $1.5\times 10^{-5}$ and $4.5\times 10^{-5}$ as the discriminator learning rate. All remaining hyperparameters stay as suggested by Mehri and Sappa [14].

We use DeOldify [25] pretrained from the official GitHub repository (https://github.com/jantic/DeOldify (accessed on 26 February 2024)) because it requires a paired dataset, which is hard to obtain. We consider its training on a larger dataset than ours as beneficial for DeOldify and thus as fair.

**Figure 2:**
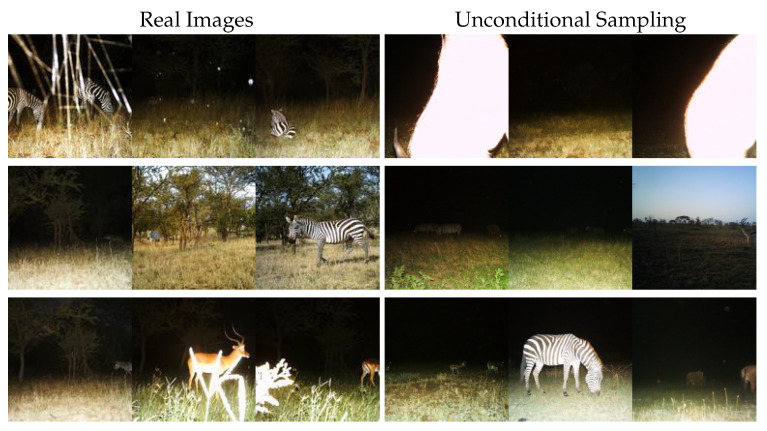
Qualitative evaluation of unconditional diffusion sampling. From left to right, we display sample images from the RGB domain of the Serengeti test dataset [8] and samples produced by the unconditional diffusion model [17].

First, we evaluate the unconditional sampling of the diffusion model and validate that the results for image synthesis of Dhariwal and Nichol [17] still hold for this dataset:

We observe in Figure 2 that the diffusion model is capable of creating diverse, realistic images. Samples such as the top-left are common for the training and test dataset as well and, therefore, are considered realistic. In Table 1, we see the diffusion network performs strongly in quantitative metrics as well. In comparison with later evaluations of our methods and CycleGAN [14], it performs at least ∼20 FID points better. Considering this, we argue this unconditional model is capable of serving as a foundation for effective colorization.

### 3.1. The Identity—Using Near-Infrared Intensities

**Figure 3:**
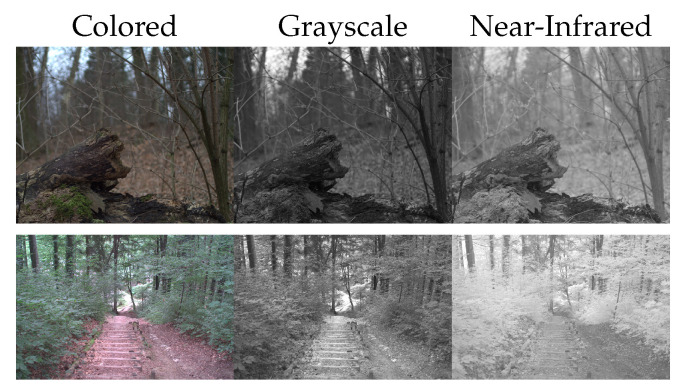
Qualitative comparison between near-infrared and visible-light images. From left to right, we display regular-colored images, intensity/grayscale images by averaging the 3 color channels and near-infrared images. All images are obtained from a dataset introduced by Brown and Süsstrunk [40], which consists of near-infrared and colored image pairs.

Physically near-infrared light is electromagnetic radiation with wavelengths between 750 nm and 1400 nm, while light from the visible spectrum lies in the range of 380 nm–780 nm [9]. The properties of an object determine which wavelength it reflects and absorbs. Hence, objects might have a strong reflectance of near-infrared light, resulting in high intensities for the observer, while absorbing more of the visible light leads to a lower intensity for the observer. In Figure 3, we show direct comparisons between the intensities of colored images (grayscale) and near-infrared images.

As visible, particularly for vegetation (last row), higher reflectance of near-infrared light in comparison to visible light is usual (Figure 3). The primary use case for near-infrared colorization in wildlife monitoring involves the colorization of nighttime images. In night images only a limited cone of illumination is available, resulting in diminished visibility of background elements such as vegetation. Consequently, one could argue in favor of disregarding the physical distinction between near-infrared and visible light and, instead, treating near-infrared images as approximations of the intensity. Song et al. [18] introduced a grayscale colorization method using diffusion models. In the context of our framework, this resolves to the identity function being the TranslateIntensity function, as shown in Section 2.2.1. Note, this is equivalent to the algorithm by Song et al. [18] for grayscale colorization.

**Figure 4:**
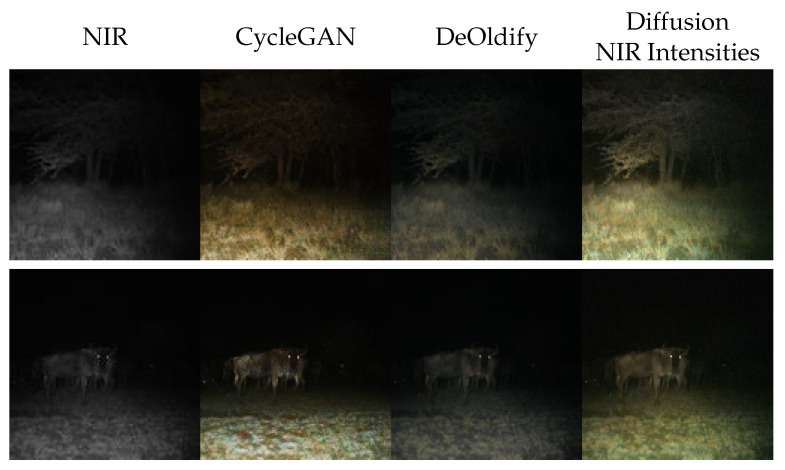
Qualitative evaluation of Iterative Seeding using NIR intensities. From left to right, we present near-infrared images from Snapshot Serengeti dataset [8]. Images generated by CycleGAN [14], by DeOldify [25], and by Iterative Seeding using the NIR implementation from Section 2.2.1.

In Figure 4, we present samples generated using this approach. We can observe that those generated images are faithful to the input image (Figure 4). Of course, this content-preservation is not a quality learned by the network but induced through our choice of intensity translation function. Most noteworthy is that the colors estimated by the diffusion model appear realistic. In comparison with DeOldify, the colorization is much more advanced. Images generated by DeOldify appear only dully colored. We argue this is because DeOldify has been trained for grayscale colorization and not for near-infrared colorization. Even though our method incorporates properties of grayscale colorization, it is more robust than DeOldify because it can estimate colors to any intensity. Qualitative weaknesses in colorization arise in comparison with CycleGAN: the diversity of images colored through this approach is low, and images appear uniformly colored. This is linked to the limitation of the approximation we made. As the intensity is strictly derived without margin, the chrominance has to be estimated for exactly this intensity. Thus, the choice of color is constrained.

Despite these minor weaknesses, the results suggest that our approximation of intensity using near-infrared intensity is reasonably accurate. It performs better than DeOldify but worse than CycleGAN, indicating a good result. As previously explained, this can be attributed to the specific condition of night images. In the majority of images, objects that reflect near-infrared light differently than visible light are typically in the background and, therefore, less illuminated. Thus, the near-infrared light approximates the visible light’s intensity. CycleGAN, on the other hand, is not restricted to changing the intensity. It is merely trained to produce invertible images and thereby can manipulate the image in favor of realism.

Like the minor qualitative weakness, we also observe the FID of this naive approach to be 12.52 FID points worse than CycleGAN’s. For NRQM, this holds too; however, only an irrelevant increase in NIQE is observable (Table 2). Additionally, we see a gap between the unconditional diffusion model and Iterative Seeding of 31.66 FID points. This indicates that this method is too restrictive to allow competitive image generation.

### 3.2. Fusing Near-Infrared and Visible Intensity through High-Pass Filtering

To address the limitation, we can draw inspiration from simple filter-based VIS-NIR fusion methods [41]. Note that the proposed framework can be considered fusioning visible and near-infrared light images in each diffusion step. Thus, it is obvious to apply techniques from the visible near-infrared fusion domain. A more refined approximation is to use only the high-frequency details of the near-infrared image while the low frequencies can still be sampled by the diffusion model, as explained in Section 2.2.2. This intuitively also provides the diffusion model freedom to sample different illuminations than those provided by the near-infrared image.

**Figure 5:**
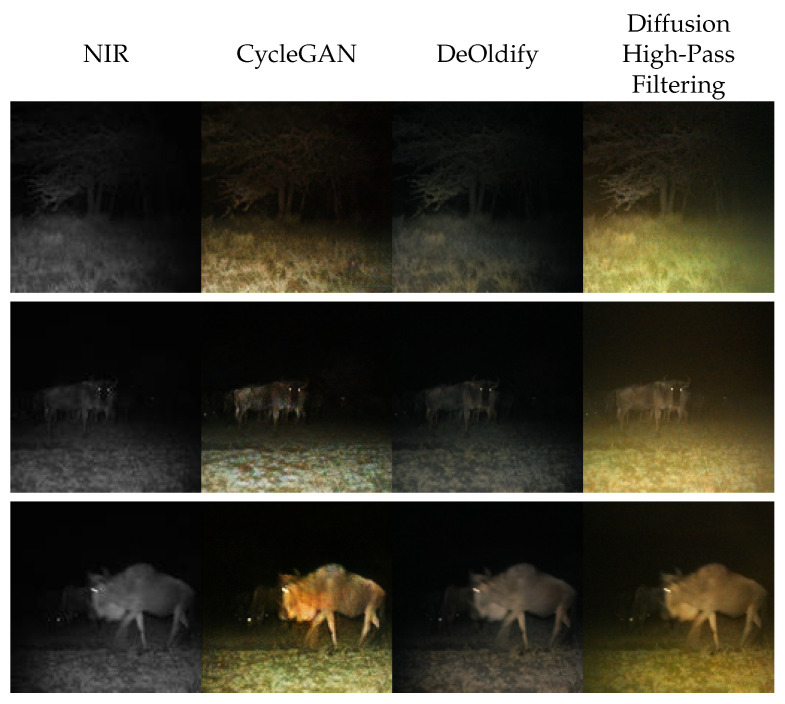
Qualitative evaluation of Iterative Seeding using high-pass filter. From left to right, we present near-infrared image from Snapshot Serengeti dataset [8], samples obtained from CycleGAN given the NIR image [14], from DeOldify [25], and from Iterative Seeding using high-pass filtering (Section 2.2.2).

In Figure 5, we evaluate our framework with this translation function. Note that we used σ=2.3 for the Gaussian filter employed in Section 2.2.2.

Samples from the diffusion model appear realistic (Figure 5). Unlike CycleGAN, which modifies smaller regions, the diffusion model modifies the global illumination of the images. In the image of the gnu (middle left), we observe that it can even qualitatively exceed the performance of CycleGAN. In that particular case, the illumination of the scene matches the illumination of the gnu, which is not the case for CycleGAN’s colorization. Additionally, Intensity Seeding using high-pass filtering performs better than DeOldify.

Moreover, it is noteworthy that the diffusion model generates a broader diversity of color schemes compared to CycleGAN: incandescent illuminations of the whole image, brown and green grass are all observable and represent the test dataset’s distribution of images well (see Figure 2 for some samples from the test dataset). However, in general, we consider CycleGAN’s colorization more realistic.

**Figure 6:**
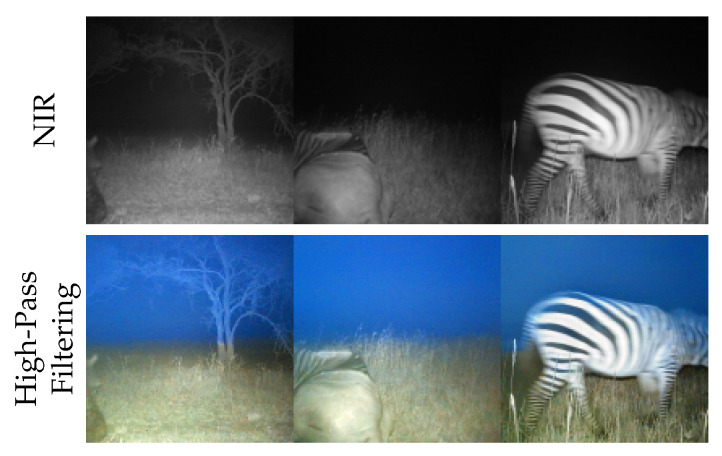
Examples of hallucinations by the diffusion model sampling. Top row shows given near-infrared images from Snapshot Serengeti dataset [8] and bottom row the samples produced by Iterative Seeding using high-pass filtering (Section 2.2.2).

Concerning content preservation, apart from global illuminations of the scene, our framework using high-pass filtering hallucinates in a few instances blue sky, as illustrated in Figure 6.

This can be attributed to a small proportion of the dataset containing images captured during dusk or dawn, where a blue sky is observable. For our test dataset, there were 56 images from 500 images that we consider to have such artifacts, which corresponds to a portion of 11.2%. Therefore, we consider CycleGAN’s content preservation stronger.

In Table 3, we provide a quantitative comparison of both methods using the FID [36].

CycleGAN outperforms this approach in terms of FID by 9.06 points, as seen in Table 3. Generally, more realistic images most likely contribute to this quantitative difference. However, this result is still 3.46 FID points better than just using the NIR intensities (Table 2). The NIQE score of the approach is 2.22 points worse while NRQM is 0.33 points worse (Table 3). This change is justified by the increase in unrealistic hallucinations, as seen in Figure 6. Thus, by employing high-pass filtering, an outcome closer to CycleGAN can be attained.

**Figure 7:**
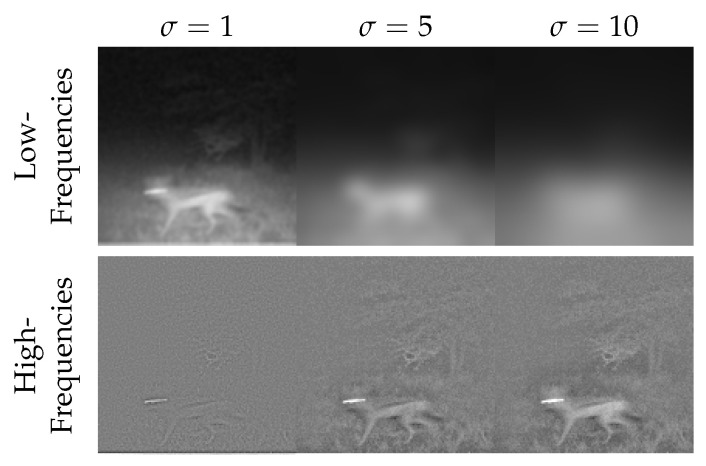
Influence of σ to low and high frequencies. In the top row, we show low frequencies of the image, and in the bottom row, we present high frequencies of the image. From left to right, we display decomposition into low and high frequencies according to Section 2.2.2 for σ=1, σ=5, and σ=10.

While introducing the hyperparameter σ in Section 2.2.2, we further want to discuss its influence on the samples. As σ controls the strength of the Gaussian filter, increasing it reduces information in the extracted low frequencies. The rise of σ leads to more information in the extracted high frequencies. We visualize this effect in Figure 7. Remember, high-frequency intensities of the near-infrared image are combined with the generated low-frequency intensities. Thus, a higher σ also corresponds to more guidance by the near-infrared image, while a lower σ results in more freedom in generation for the model.

**Figure 8:**
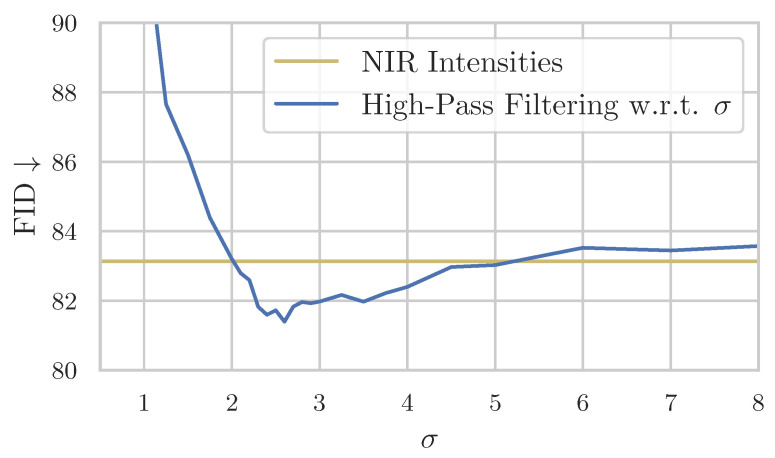
Influence of hyperparameter σ on the FID. On the y-axis, the FID (lower is better) and on the x-axis σ with which was sampled. The blue line shows the FID of Iterative Seeding using high-pass filtering with respect to σ (Section 2.2.2), and the yellow line shows the FID of Iterative Seeding using NIR intensities (Section 2.2.1) for comparison. All FID scores are obtained using the same random seed to reduce outliers.

In Figure 8, we visualize how σ affects the sampling quality in terms of FID. We sample with the same seed, with the intention to have as few influences of randomness as possible. It is observable that samples with a σ of less than 1 are quantitatively worse (Figure 8). Our hypothesis is that, at this stage, some guidance is provided, but it is insufficient, making sampling within these boundaries too challenging for the model. At σ of 2.3, the minimum FID is reached. At this point, an adequate amount of guidance is provided for the model to perform reasonable sampling of color, without imposing too many constraints limiting the model’s generation freedom.

For higher σ, the diffusion model receives more guidance, and consequently, it has less freedom for its generation process. Thus, the FID rises. On the other hand, we observe by comparing manually that the proportion of hallucinated blue sky becomes less frequent.

Hence, we regard σ as a hyperparameter that controls the compromise between realism and content preservation.

### 3.3. The Potential of Diffusion-Based Near-Infrared Colorization—CycleGAN as Intensity Translation Function

We note that our existing translation function implementations do not result in the diffusion model surpassing CycleGAN either quantitatively or qualitatively. However, the translation function we employed was of a trivial nature and did not exhaust the research results from the near-infrared visible fusion field (e.g., see advanced approaches in [20]). Nevertheless, it does generate realistic images (Figure 5), achieves FID scores close to CycleGAN (Table 3), and performs better than the identity (Table 2).

**Figure 9:**
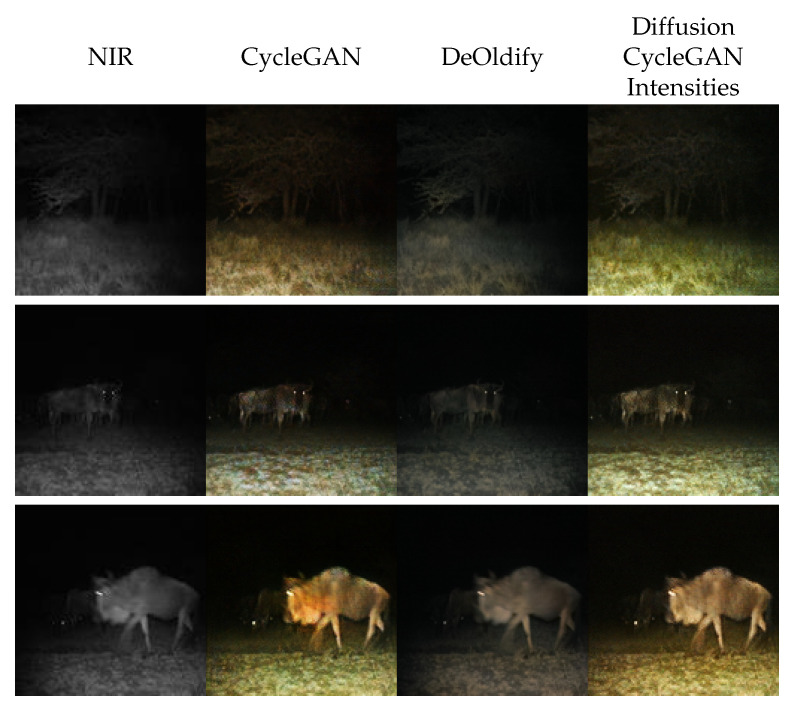
Qualitative evaluation of Iterative Seeding using CycleGAN intensities. From left to right, we present near-infrared images from Snapshot Serengeti dataset [8], images generated by CycleGAN [14], by DeOldify [25], and by Iterative Seeding using CycleGAN intensities.

This indicates there is unexhausted potential for this framework. To prove this, we use a translation function known to generate good results: CycleGAN itself. Precisely, this translation function evaluates the trained CycleGAN colorization for the NIR image and uses only the intensity of this image as input for the diffusion model. Therefore, our model still estimates the color but uses the intensity generated from CycleGAN. In Figure 9, we present samples using this translation function.

We observe the diffusion model does not only effectively colorize these images but also surpasses CycleGAN in terms of color selection Figure 9. Unlike CycleGAN, the gnu generated by the diffusion model has matching illumination to the rest of the image.

In quantitative terms, this approach performs 4.47 FID points better than CycleGAN itself (Table 4). The same holds for the NIQE and NRQM metric which indicates a slightly improved realism. Because the model does not only achieve a similar score to CycleGAN using its intensity but also exceeds CycleGAN’s score, the potential of diffusion-based colorization is shown. CycleGAN generating the intensity by itself, does not generate colors as good as the diffusion model does.

## 4. Discussion

We introduce a framework for diffusion-based NIR image colorization. It abstracts the translation of intensity and utilizes diffusion models for the effective colorization.

### 4.1. Implementations of Intensity Translation

We propose and evaluate three variants for the implementation of the intensity translation function.

1. We demonstrate directly using the NIR intensity, effectively representing an identity function. This simple implementation creates a basic colorization, but the diffusion network is too restricted by the NIR intensity, yielding suboptimal realism and FID scores. Even though this colorization is not specialized for near-infrared colorization, it performs more robustly than other grayscale colorization methods, as the comparison with DeOldify suggests.
2. Because the framework can be considered iteratively fusing near-infrared and visible light, we draw inspiration from the VIS-NIR fusion research domain. A key observation made in this field is to use the high-frequency intensities of the near-infrared image and fuse them with the low-frequency intensities of the visible-light image. We apply this insight to our framework in a trivial implementation using a Gaussian filter. An improvement in comparison with just using the NIR images as intensities is observed quantitatively and qualitatively, resulting in an FID score close to our baseline CycleGAN. Additionally, with the ClipFID, a different FID variant not relying on the Inception model and the ImageNet dataset [42], we achieve a score of 7.87 using this translation method compared to CycleGAN 9.15. However, because the ClipFID is not established as a comparison metric, this result has to be treated carefully. Even though this implementation is far from exhausting results from the VIS-NIR fusion domain, it achieves scores close to our baseline, suggesting a more sophisticated implementation can achieve even better results.
3. Finally, we evaluate CycleGAN itself as an intensity translator. Using intensities generated by CycleGAN our framework outperforms CycleGAN quantitatively as well as qualitatively. This indicates the potential of our framework for sophisticated translation functions and diffusion-based NIR colorization in general. Considering this potential, we show that our framework reduces NIR colorization to visible near-infrared fusion, a simpler problem.

### 4.2. Colorizing NIR Images for Animal Detection

The integration of camera trapping with artificial intelligence (AI), particularly leveraging deep learning methodologies, represents a significant advancement in wildlife research and conservation [3,7,11,12]. Nevertheless, numerous deep learning models are optimized for and perform better with colored images, akin to human perception [10]. To explore this phenomenon, we assess the efficacy of the proposed diffusion-based NIR colorization technique in enhancing image classification within camera trap datasets.

We utilize a subset of randomly selected night images in near-infrared (NIR) from the Snapshot Serengeti dataset [8]. This subset is divided into 4000 images for training and 500 each for the validation and test datasets. Subsequently, all 5000 images are colorized using each method outlined. For every method, we fine-tune a ResNet50 [24] classifier pretrained on ImageNet-21K [43] on the training dataset derived from the respective method. We use a cross-entropy loss with an Adam optimizer (β=(0.9,0.999) and $\mathrm{lr}=10^{-4}$). Finally, we evaluate the model on the test dataset acquired from each method. This experiment is repeated five times, and the average over all five accuracies is used. Additionally, we repeat this evaluation for a ResNet using the same pretrained weights but with freezing all layers except the final classification layer. We argue this score is a measurement for content preservation, as the network can only classify accurately if the relevant content is translated.

Table 5 displays the classification accuracies of various methods. In both the frozen and unfrozen scenarios, both CycleGAN and our method utilizing its intensities achieve similar accuracies. However, our methods, employing near-infrared intensities or employing high-pass filtering, outperform both CycleGAN and the diffusion approach utilizing CycleGAN’s intensities. Specifically, for the unfrozen network, and even more significantly for the frozen network, our methods achieve an accuracy improvement of approximately 6.83 percentage points.

However, when unfrozen, none of the methods used yield improved classification accuracy compared to directly utilizing near-infrared intensities. Conversely, for the frozen network simulating few-shot learning, using high-pass filtering results in accuracies rivaling those achieved by direct NIR intensity utilization. However, the improvement of our variant is only that marginal such that it lies in the standard deviation of both accuracies. This could be attributed to the robustness of ResNet [24], a powerful deep learning approach for object classification, which is not specialized for colored images and can handle various input formats adeptly. When the network’s backbone is frozen the feature extraction of the network is settled, and only the classification using those features is trained. Thus, the accuracy benefits from colored images more. The lower accuracies in general result from the fact that the network is also in general more restricted to adjust to the given images.

### 4.3. Colorizing NIR Images for Animal Detection Explainability

We employ the AI-based ResNet [24] approach for visual animal detection. An improvement of the classification accuracy by using colorized images can only marginally be observed.

However, we have to take into account user acceptance and explainability of AI-based approaches to animal detection. Many AI-based systems and especially deep learning (DL) methods (like ResNet) are black-box models that are extremely hard to explain and to understand even by domain experts [44]. Explainable AI (XAI) and explainable machine learning refer to AI approaches that allow users to retain understanding and acceptance. Many AI-based and especially DL-based approaches to object recognition in general and animal recognition in particular are so-called data-centric AI (DCAI) methods. DCAI shifts the focus from hand-crafted model building to curating high-quality, consistently annotated training datasets.

Therefore, understanding and accepting AI-based animal recognition heavily relies on the understanding and acceptance of the employed training datasets, i.e., the training images. Using NIR images for training AI-based systems for animal detection decreases user acceptance because their appearance does not match with human perception [10,21]. Thus, our approach to NIR image colorization improves the utilization of NIR images and NIR video clips for education, promotion, and funding acquisition.

### 4.4. Novelty and Scientific Relevance

The novelty of our approach lies primarily in the iteration step during inference, where we merge the current sample with the given image. Unlike existing methods, we differentiate between merging chrominance and intensity components. Specifically, we extract chrominance from the input image, and we introduce a novel abstraction of intensity merging to suit diffusion models for near-infrared colorization. This innovation is motivated by the three distinct implementations we present, each demonstrating the significance of the performance of this crucial step. From a scientific standpoint, our framework serves as a general framework for diffusion-based near-infrared colorization techniques.

By showcasing the superiority of diffusion-based methods over GAN-based approaches, we contribute to the near-infrared colorization research. Moreover, we present an intersection of near-infrared colorization, near-infrared-visible fusion, and diffusion models, thus contributing to the advancement of these interconnected fields.

### 4.5. Limitations and Challenges

One limitation of a diffusion model approach is that it requires much more computational resources in training and inference than CycleGAN. This manifests also in the training and sampling durations: although we trained CycleGAN over the course of two days and inference is a matter of mere seconds, the diffusion network required two weeks for training, and generating 500 samples took approximately 40 min on an NVIDIA RTX A5000.

One challenge of our approach is the design of the intensity translation and merging process. It has to incorporate enough information from the given image to preserve the content while it should not use too much to generate a realistic RGB image. We present three example methods for this process and prove its potential; however, future research is needed to find an optimal method. Inspiration can be drawn from further VIS-NIR fusion research; alternatively, different machine learning techniques can also be applied to solve this subproblem (see Section 5.1).

Another challenge faced during this study is the evaluation process. As we focus on unpaired image translation and only have such a dataset, paired evaluation techniques such as mean-squared distances and SSIM can not be used. Instead, we solved this using the unpaired dataset distance FID [36], measures like the classification accuracy and, lastly, no-reference image quality assessment metrics NIQE [38] and NRQM [39]. Although combining all these metrics does provide a robust evaluation, a paired dataset for evaluation and unpaired for training would be optimal (see Section 5.1).

## 5. Conclusions

This study presents the first framework utilizing diffusion models for the colorization of near-infrared (NIR) images. We show that the effectiveness of colorizing NIR images is primarily controlled by the translation of the intensities of near-infrared light to those of visible light.

1. Iterative Seeding on NIR intensities (ISNIR);
2. Iterative Seeding using high-pass filtering (ISHP);
3. Iterative Seeding using CycleGAN intensities (ISCG).

Inspired by research from visible near-infrared fusion, we have shown that even employing ISHP as a simple algorithm for translating NIR intensities achieves FID scores close to the GAN baseline. Thus, we establish a connection between near-infrared colorization, diffusion models, and visible near-infrared fusion.

Furthermore, our framework is shown to outperform the GAN baseline with the ISCG implementation as indicated by decreasing FID, NIQE, and NRQM values.

In general, our method bridges the domain gap between near-infrared and colored images and addresses challenges of near-infrared colorization including the lack of paired training data, as well as the different reflectance properties of near-infrared and visible light.

### 5.1. Future Work

For future research, several variations to our proposed framework are feasible. First, recent advances in latent diffusion models (LDMs) [45] could potentially allow sampling of higher resolutions, such as 1024×1024, increase the sampling speed, and therefore mitigate the drawbacks of our approach in a practical implementation. Deterministic samplers such as DDIM [46] or using a formulation based on ordinary differential equations, as demonstrated by Song et al. [18], could contribute to this.

One potential direction for future research involves exploring more sophisticated approaches from the VIS-NIR fusion field for implementing our framework [20]. Additionally, a hyperparameter scaling the score function, as done by Dhariwal and Nichol [17] for classifier guidance, could allow a built-in approach for controlling the trade-off between realism and content preservation.

Our method, although presented in the context of wildlife monitoring, may not be restricted to it. Therefore, an evaluation of other datasets and application contexts, such as in driver assistance systems could reveal valuable insights. A paired dataset for evaluation and unpaired for training would additionally contribute to an optimal study evaluation.

Finally, using high-frequency details of the near-infrared image to enhance the colored image is a concept not reserved for diffusion models, e.g., introducing a loss between the high-frequency intensities of the generated and given images could potentially benefit CycleGAN, too.

## Figures and Tables

**Table 1:** Quantitative evaluation of unconditional diffusion sampling. The unconditional diffusion model [17] trained and evaluated on the Snapshot Serengeti dataset [8] containing only night NIR and RGB images. We compare the FID calculated between the test dataset and the generated images.

Model	FID ↓
Unconditional Diffusion Model [17]	55.01

**Table 2:** Quantitative evaluation of Iterative Seeding Using NIR Intensities. Samples of CycleGAN [14], DeOldify [25], and Iterative Seeding on NIR intensities (Section 2.2.1) are generated. The FID [36] is calculated by comparing the test dataset and the set of generated samples.

Model	FID ↓	NIQE ↓	NRQM ↑
CycleGAN [14]	74.15	14.06	5.45
DeOldify [25]	104.07	17.93	4.41
Iterative Seeding Using NIR Intensities	86.67	14.08	4.93

**Table 3:** Quantitative evaluation of Iterative Seeding using high-pass filter. We compare CycleGAN [14], DeOldify [25], and Iterative Seeding using high-pass filtering (Section 2.2.2) on the Snapshot Serengeti dataset [8]. The FID [36] is calculated between the test dataset and the generated images.

Model	FID ↓	NIQE ↓	NRQM ↑
CycleGAN [14]	74.15	14.06	5.45
DeOldify [25]	104.07	17.93	4.41
Iterative Seeding High-Pass Filtering	83.21	16.28	4.74

**Table 4:** Quantitative evaluation of Iterative Seeding using CycleGAN intensities. Samples are obtained from CycleGAN [14], from DeOldify [25], and from Iterative Seeding using CycleGAN intensities. The FID [36] is calculated by comparing the test dataset and the set of generated samples.

Model	FID ↓	NIQE ↓	NRQM ↑
CycleGAN [14]	74.15	14.06	5.45
DeOldify [25]	104.07	17.93	4.41
Iterative Seeding CycleGAN Intensities	69.68	13.47	5.47

**Table 5:** Quantitative evaluation of classification using colorized images Comparison of FID and classification accuracy on images from the NIR dataset, samples from CycleGAN [14] and Iterative Seeding using all presented implementations. The FID [36] is calculated by comparing the test dataset and the set of generated samples. Classification accuracy is obtained by training a ResNet classifier for each method and calculating the accuracy afterward on a test dataset. We either train all layers (non-frozen) or freeze all but the final classification layer (frozen). The accuracy is averaged over 5 runs, and σ displays the corresponding standard deviation.

		Non-Frozen		Frozen	
Model	FID ↓	Accuracy↑	σ	Accuracy ↑	σ
NIR	-	0.7276	0.0124	0.3628	0.0105
CycleGAN [14]	74.15	0.5341	0.0146	0.3447	0.0123
NIR Intensities	86.67	0.6024	0.0102	0.3554	0.0213
High-Pass Filtering	83.21	0.6078	0.0119	0.3681	0.0090
CycleGAN Intensities	69.68	0.5314	0.0180	0.3367	0.0116

## Data Availability

Source code for downloading the evaluation dataset, for our evaluations, and for our proposed framework is available at https://github.com/aykborstelmann/nir-coloring (accessed on 26 February 2024). Our dataset is based on the Snapshot Serengeti dataset [8], which is openly available, as well at https://doi.org/10.5061/dryad.5pt92 (accessed on 26 February 2024).

## Abbreviations

The following abbreviations are used in this manuscript:

NIR	near-infrared
RGB	red, green, blue (color channels)
VIS-NIR fusion	visible-near-infrared fusion
GAN	generative adversarial network
ILVR	iterative latent variable refinement
FID	Fréchet inception distance
CNN	convolutional neural network
